# Excellent Response with Ado-Trastuzumab Emtansine in a Patient with Relapsed Metastatic Breast Cancer Presenting with Pulmonary Lymphangitic Carcinomatosis

**DOI:** 10.7759/cureus.1473

**Published:** 2017-07-14

**Authors:** Zhou Yu, Shobana Sankar, Marianne Huben

**Affiliations:** 1 Department of Hematology and Oncology, William Beaumont Hospital, Oakland University William Beaumont School of Medicine; 2 Department of Internal Medicine, William Beaumont Hospital, Oakland University William Beaumont School of Medicine; 3 Department of Hematology and Oncology, William Beaumont Hospital, Oakland University William Beaumont School of Medicine

**Keywords:** breast cancer, trastuzumab, t-dm1, her2

## Abstract

In breast cancer, aggressive tumor biology and the corresponding poor prognosis is associated with amplification or overexpression of the human epidermal growth factor receptor 2 (HER2). Trastuzumab has significantly changed the natural history of HER2-positive breast cancer. However, resistance to trastuzumab remains a substantial clinical problem. Ado-trastuzumab emtansine (T-DM1), an antibody-drug conjugate, has demonstrated impressive results in second- or later-line treatment of HER2-positive breast cancer. We report a case of 43-year-old female with previously trastuzumab-treated HER2-positive breast cancer relapsed with pulmonary lymphangitis carcinomatosis that responded dramatically to T-DM1 therapy.

## Introduction

Breast cancer remains the most common cancer diagnosed in women. In 2017, 318,580 new breast cancer cases and 40,610 breast cancer deaths were projected to occur in women in the United States [1]. Human epidermal growth factor receptor 2 (HER2) is a transmembrane tyrosine kinase receptor that regulates cellular growth and proliferation in epithelial cells. Historically, patients with HER2-positive breast cancer had an aggressive tumor biology which is associated with extremely poor prognoses. Now, with anti-HER2 therapy, tumor response and patient survival have dramatically improved [2]. In this paper, we present a case of relapsed HER2-positive breast cancer that showed a dramatic response with anti-HER2 targeted therapy.

## Case presentation

A 43-year-old female was diagnosed with stage IIIA invasive ductal carcinoma of right breast (T2N1M0, stage IIIA) two years ago. After completing neoadjuvant chemotherapy (six cycles of pertuzumab, trastuzumab, docetaxel and carboplatin), she underwent a curative resection for the breast cancer. Final pathology showed marked reduction in tumor burden with only a 5% residual tumor in the tumor bed. There was a single focus of lymphovascular invasion and the surgical margins were uninvolved. Immunohistochemistry revealed positive staining for the estrogen receptor (ER, 90%), progesterone receptor (PR, 2%), and HER2 (score 3+). She then underwent a course of external beam radiation therapy to the right breast and supraclavicular region. She was started on endocrinology therapy with tamoxifen and completed a year of trastuzumab treatment. She remained disease-free until two years later, when she presented with a persistent non-productive cough for two months; during the period, the patient had thought the cough was related to allergies. The patient underwent a routine exam with her surgeon and was found to have non-tender enlarged neck lymph nodes. An ultrasound-guided biopsy of the left neck lymph node revealed adenocarcinoma consistent with breast origin, with immunophenotype positive for ER (36%), negative for PR, and positive for HER2. The patient was referred back to oncology for additional evaluation.

**Figure 1:**
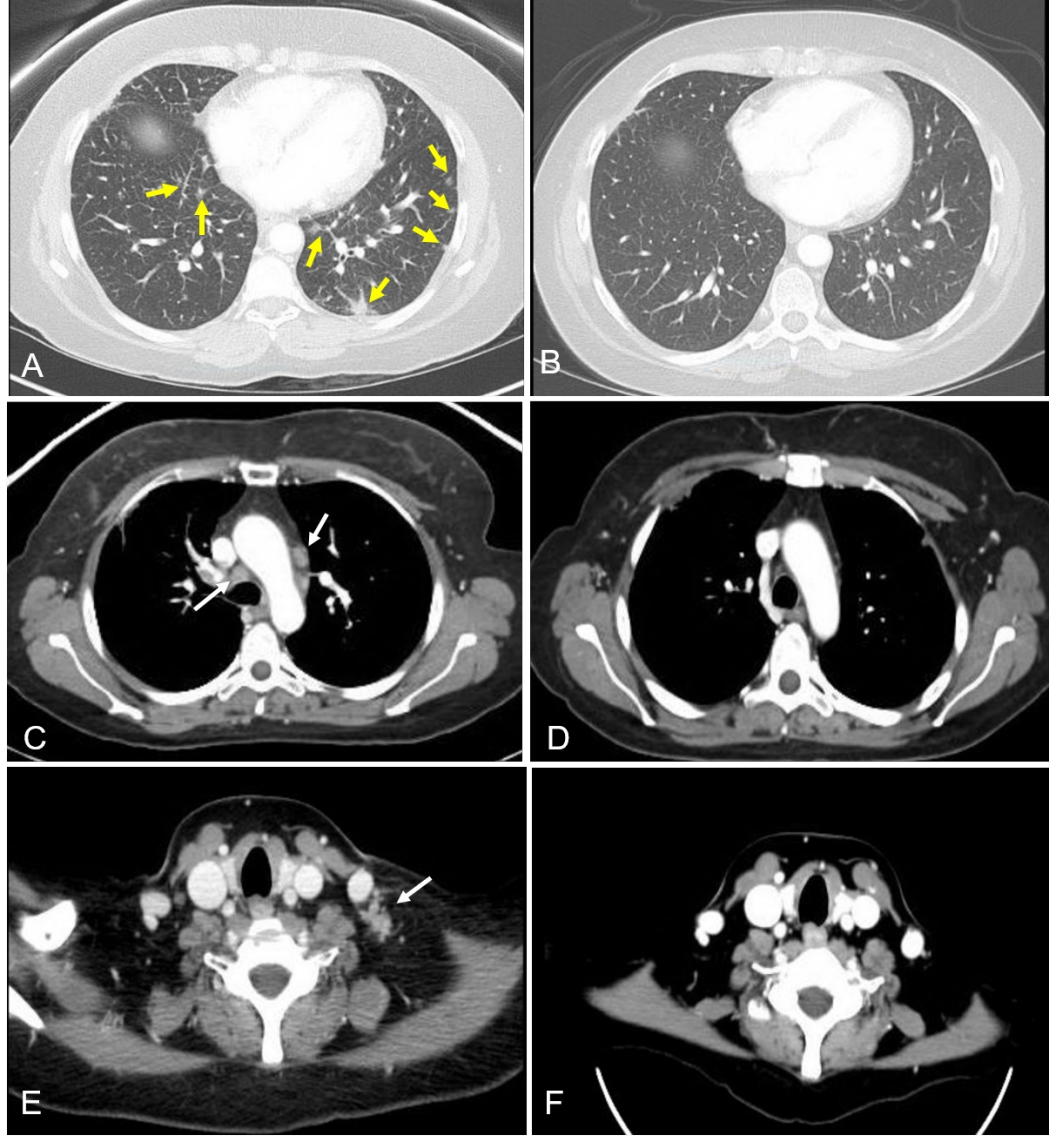
Comparison of computed tomography images of lung and neck before and after treatment Pulmonary lymphangitic carcinomatosis manifested with numerous, ill-defined, bilateral peribronchovascular nodular opacities; (A) yellow arrows pointing at the nodular thickening of interlobular septa and several lateral and posterior nodules. A follow-up computed tomography scan after three cycles of ado-trastuzumab emtansine treatment showed resolution of most nodules (B). Marked mediastinal (C) and cervical (E) lymphadenopathy before treatment is shown (arrows). A follow-up computed tomography study after treatment showed completely resolved lymphadenopathy (D, F).

Computed tomography (CT) demonstrated numerous ill-defined peri-bronchial lung nodules which were consistent with lymphangitic metastatic carcinoma pattern (Figure 1A). In addition, there were conglomerate supraclavicular, cervical, and mediastinal lymphadenopathy (Figure 1C, 1E). Restaging images also demonstrated brain and bone metastatic disease. Her cough had progressed rapidly and became intractable during the ten-day workup period. The patient was started on ado-trastuzumab emtansine (T-DM1) at 3.6 mg/kg intravenously every three weeks. In addition, she also received whole-brain radiation therapy (WBRT). She tolerated the treatments well without significant side-effects. Her intractable cough completely resolved within two weeks of starting the treatment and her enlarged neck lymph nodes dramatically shrank after one cycle of T-DM1 treatment. After three cycles of treatment, follow-up CT images showed the disappearance of the majority of lung nodules (Figure 1B) and complete resolution of her mediastinal and neck lymphadenopathy (Figure 1D, 1F). Her metastatic bone and brain lesions remained stable. The sequence of events are summarized in Table 1.  

**Table 1:** Summary of the sequence of events CT: computed tomography; T-DM1: Ado-trastuzumab emtansine

Timing of the events	Diagnosis	Treatments	Outcome
September, 2014	Right breast invasive ductal carcinoma (T2N1M0, stage IIIA)	Neoadjuvant chemotherapy followed by surgery, followed by radiation to right breast and supraclavicular region. She also completed one year of trastuzumab and was also started on tamoxifen	Remission
August, 2016	Relapsed metastatic breast cancer with pulmonary carcinomatosis, CNS, and bone metastasis	T-DM1 treatment	Near complete resolution of pulmonary carcinomatosis
January, 2017	Restage CT scan of chest	After three cycles of T-DM1 treatment	Disappearance of the majority of lung nodules
March, 2017	Restage CT scan of neck and chest	After six cycles of T-DM1 treatment	Complete resolution of mediastinal and neck lymphadenopathy

At the time of this report, the patient has remained on T-DM1 treatment for eight months, and she has been free from disease progression. She will be maintained on T-DM1 until disease progression or unacceptable toxicity. Written consent was obtained from the patient for the publication of this case report and accompanying images. No identifying patient information was disclosed in this paper. 

## Discussion

Metastatic cancer with pulmonary lymphangitic carcinomatosis (PLC) is characterized by the diffuse spread of the tumor to the pulmonary lymphatic system. It is a type of visceral crisis and is mostly seen in adenocarcinomas originating from breast (33%), stomach (29%), lung (17%), pancreas (4%) and prostate (3%) [3]. Non-productive cough and dyspnea, as seen in our patient, are the most common presenting symptoms of PLC. In general, diagnosis may be delayed due to the occurrence of non-specific symptoms and symptoms with characteristics similar to sarcoidosis and interstitial lung diseases. Chest radiographs appear normal in 30%–50% of patients with histologically proven disease. Computed tomography (CT) characteristics include nodular thickening of interlobular septa, peribronchovascular interstitium with polygonal arcades with thickened limbs from thickened septa of adjacent lobules, pleural effusion, and lymphadenopathy [3]. In historical case series, the prognosis of patients with PLC is very poor with an average survival of only three months [3]. HER2-positive breast cancer is associated with a more aggressive clinical phenotype and historically portends poor prognosis. Over the last two decades, therapies directed against HER2 have transformed HER2-positive breast cancer into a highly treatable disease. Trastuzumab is a recombinant humanized monoclonal antibody that inhibits HER2 signaling. Data from multiple randomized clinical trials show that trastuzumab dramatically improves response rate, progression-free survival (PFS), and overall survival (OS) in patients with early stage or metastatic HER2-positive breast cancer [2]. However, despite this progress, a proportion of patients with early-stage disease still suffer from recurrence after neoadjuvant or adjuvant trastuzumab treatment and most metastatic patients develop progression on treatment.

T-DM1 is a novel anti-HER2 therapy agent which has showed impressive efficacy and improved outcome in HER2-positive breast cancer that is resistant to trastuzumab. It is a conjugate of the humanized anti-HER2 antibody trastuzumab and the derivative of maytansine (emtansine, DM1), a potent microtubule-disrupting agent. Upon binding to the HER2 receptor, T-DM1 is internalized via receptor-mediated endocytosis, and cytotoxic DM1 is subsequently released intracellularly by lysosomal degradation. While trastuzumab is utilized to deliver cytotoxic agent emtansine highly selective to the HER2 expressed cancer cell, T-DM1 also retains the trastuzumab’s suppressive activity of HER2 signaling and antibody-dependent cell-mediated cytotoxicity [2]. In a Phase III EMILIA study, T-DM1 prolonged PFS (9.6 vs 6.4 months) and OS (30.9 vs 25.1 months) compared to lapatinib and capecitabine in metastatic HER2-positive breast cancer previously treated with trastuzumab and a taxane. The objective response rate was also improved (43.6% vs 30.8%) and the median duration of response was prolonged in the T-DM1 arm (12.6 months vs 6.5 months) [4]. The most common grade 3 and 4 adverse events with T-DM1 were thrombocytopenia (12.9%) and elevated liver transaminase (7.2%), compared to hand foot syndrome (16.4%) and diarrhea (20.7%) in the control arm. In February 2013, based on EMILIA results, T-DM1 was approved by the Food and Drug Association (FDA) for the treatment of HER2-positive pre-treated metastatic breast cancer. This is the first targeted chemotherapy treatment for breast cancer. Another randomized phase III trial, TH3RESA study, compared T-DM1 to the treatment of the physician’s choice in a patient who progressed on two or more anti-HER2 treatments. Median PFS in the T-DM1 arm was nearly double that of patients in the control arm (6.2 vs 3.3 months), and median OS were significantly longer in T-DM1 arm (22.7 months vs 15.8 months). Severe treatment-related toxicities were more frequent in the physician’s treatment choice arm [5-6]. These two phase III trials solidified the role of T-DM1 as a standard choice in second- or later-line treatment in metastatic HER2 positive breast cancer. The use of T-DM1 in a front-line setting was investigated in the phase III MARIANNE study. T-DM1 demonstrated non-inferior PFS compared to trastuzumab and taxane and is considered as an alternative drug for patients who are not suitable for standard regimen [7]. 

## Conclusions

Trastuzumab has significantly changed the natural history of HER2-positive breast cancer. However, resistance to trastuzumab remains a substantial clinical problem. T-DM1, an antibody-drug conjugate, has demonstrated impressive results in second- or later-line treatment of HER2 positive breast cancer. Our case stands as an example of an exceptional response to the T-DM1 treatment in a previously trastuzumab-treated patient who presented with extremely aggressive pulmonary lymphangitic metastasis. 
